# Supplementary material for: Experimental warming decreases arbuscular mycorrhizal fungal colonization in prairie plants along a Mediterranean climate gradient
Source: PeerJ. 2016 Jun 1;4:e2083. doi: 10.7717/peerj.2083 (PMC4893335; doi:10.7717/peerj.2083)
Supplement: Supplemental Information 1 [file peerj-04-2083-s001.docx]

**Supporting Information**

**Figure S1.** Nutrient availability measured with anion-cation exchange probes for inorganic P (a), total inorganic N (b), and ratio of the two nutrients (c) for control and heated treatments within each site. Different letters indicate significant differences among sites. Asterisks represent significant differences between control and heated treatments (*** = *P* < 0.001, ** = *P* < 0.01, * = *P* < 0.1). Error bars are represented as +/- one SE.


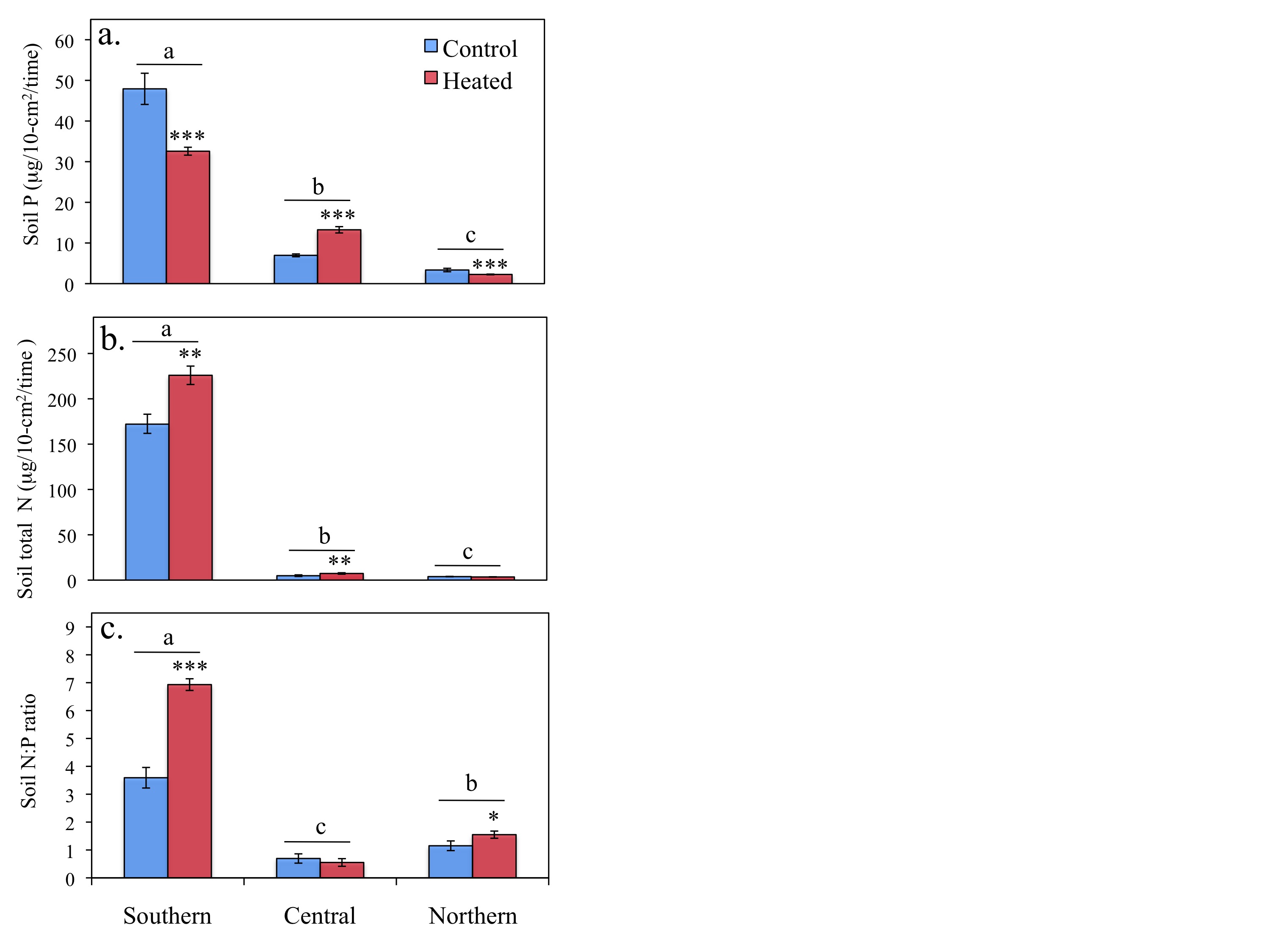


**Figure S2.** Percentage of P (a), total N (b), and the N to P ratio (c) of plant material across sites and species. Different letters indicate significant differences among sites within a species. Asterisks represent significant differences between control and heated treatments (*** = *P* < 0.001, ** = *P* < 0.01, * = *P* < 0.1). Error bars are represented as +/- one SE. *Plectritis* lacks errors bars for treatments with replicates of N=1. Dashed line represents the approximate point at which P and N are co-limited.


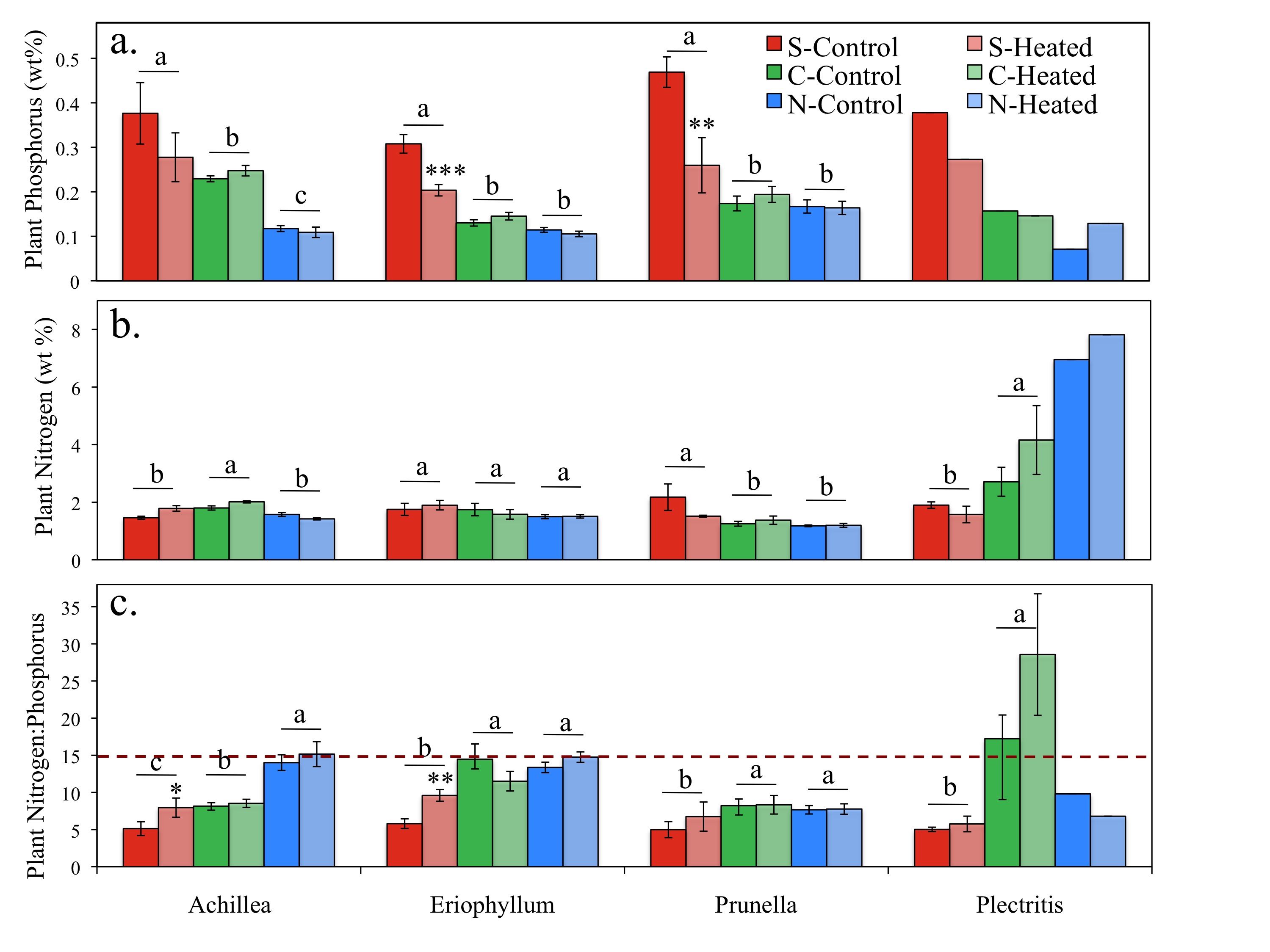


**Figure S3.** Phosphorus structural equation model including data from all sites and species. Each box represents a variable in the model, and while the number above each arrow represents the value of the standardized path coefficients. The width of each arrow corresponds with the magnitude of the path coefficient, solid lines indicate positive effects, and dashed lines indicate negative effects. Path coefficients that were not significant to *P* < 0.1, are not shown. The italicized number above each box represents the total explained variance (R^2^) of each variable.

**Figure S4.** Southern N:P structural equation model including only data from the southern site, but all species. Each box represents a variable in the model, and while the number above each arrow represents the value of the standardized path coefficients. The width of each arrow corresponds with the magnitude of the path coefficient, solid lines indicate positive effects, and dashed lines indicate negative effects. Path coefficients that were not significant to *P* < 0.1, are not shown. The italicized number above each box represents the total explained variance (R^2^) of each variable.


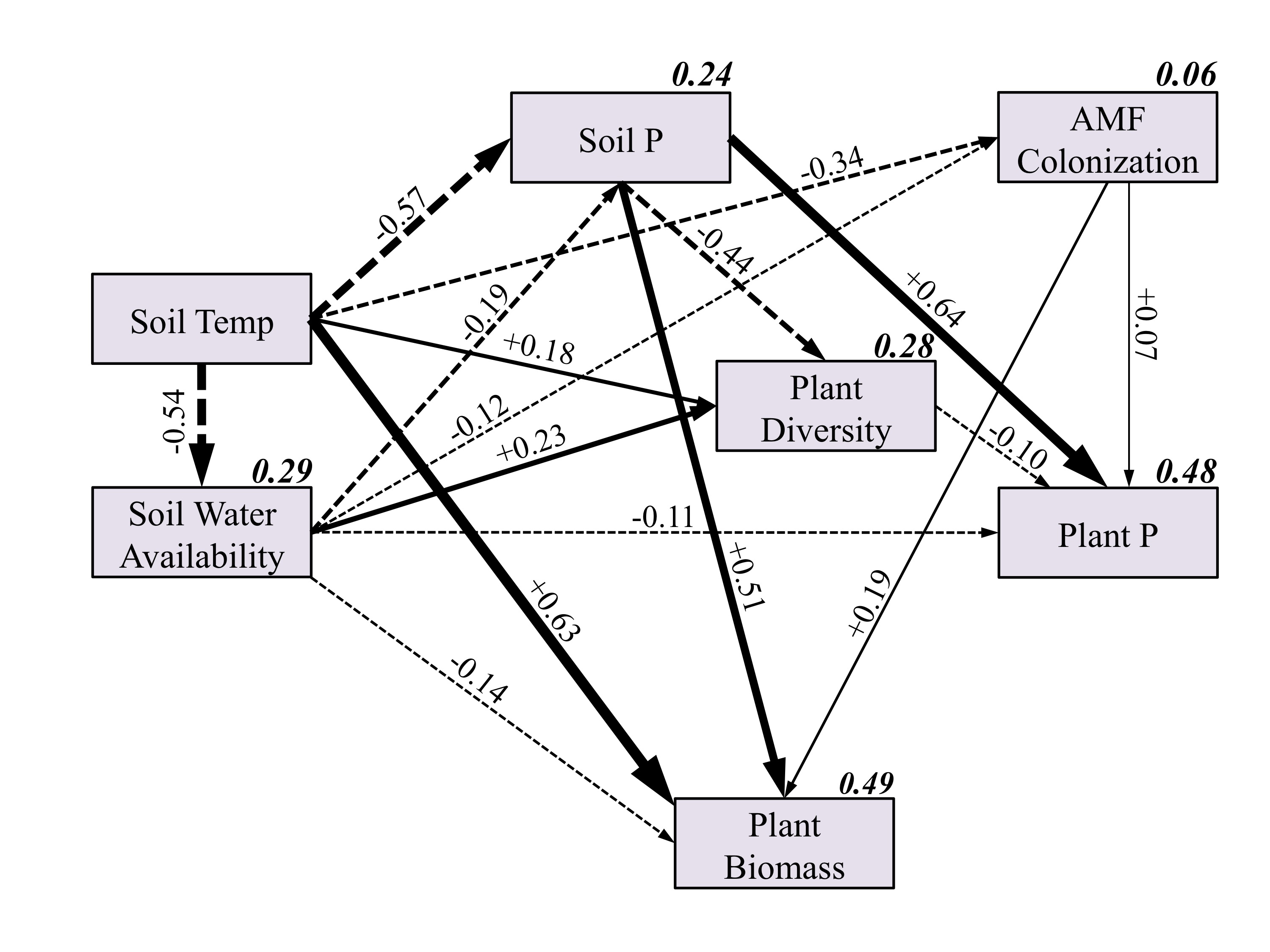

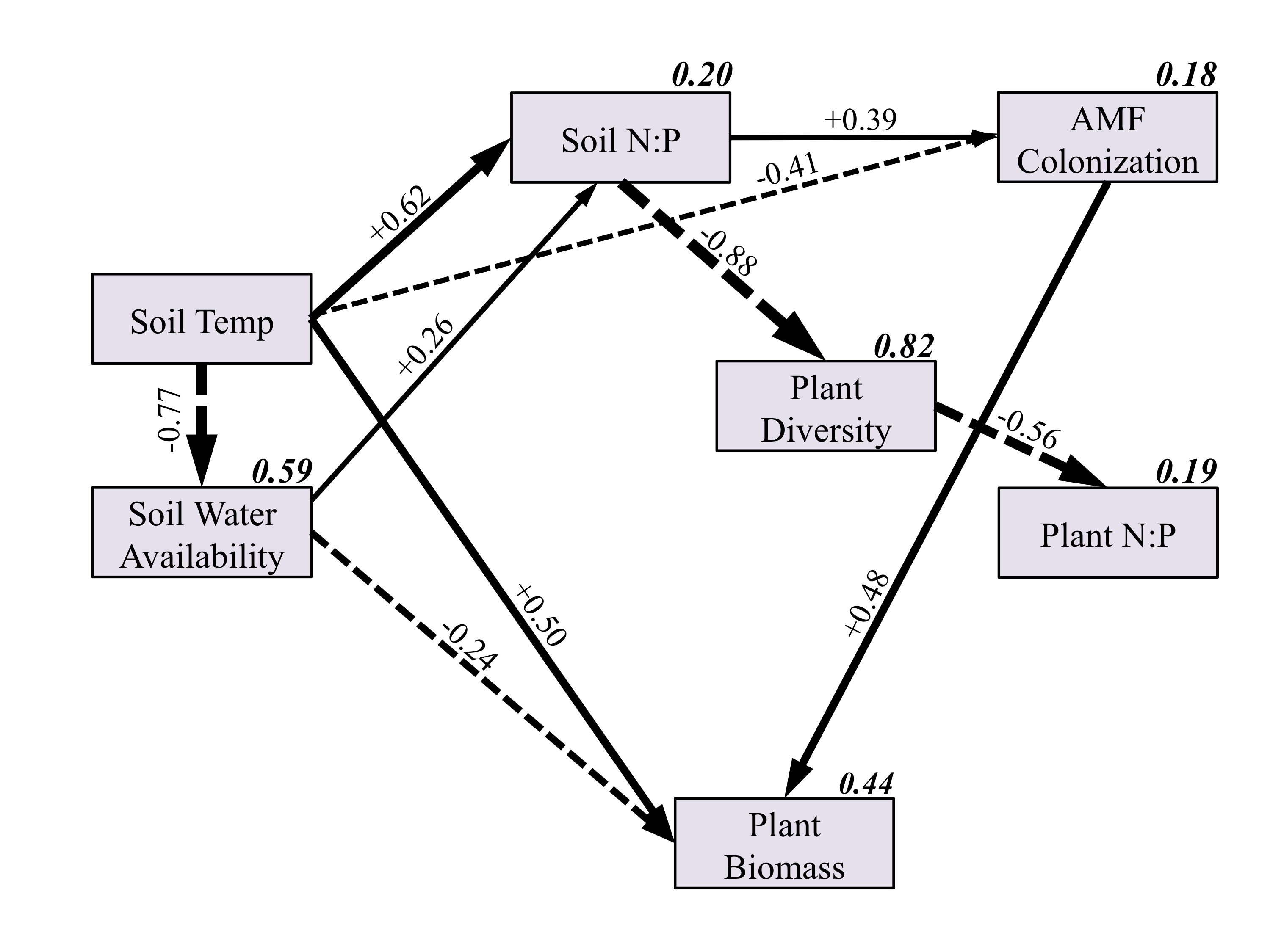


**Figure S5.** AMF species from the host plant *Eriophyllum lanatum* identified by the trail *Illumina* sequencing run grouped by family. Only 1% of original sequence data were matched to AMF, though these species range across most families of AMF.

**Table S1.** Species of AMF identified from host species *Eriophyllum lanatum* in the *Illumina* sequencing run.


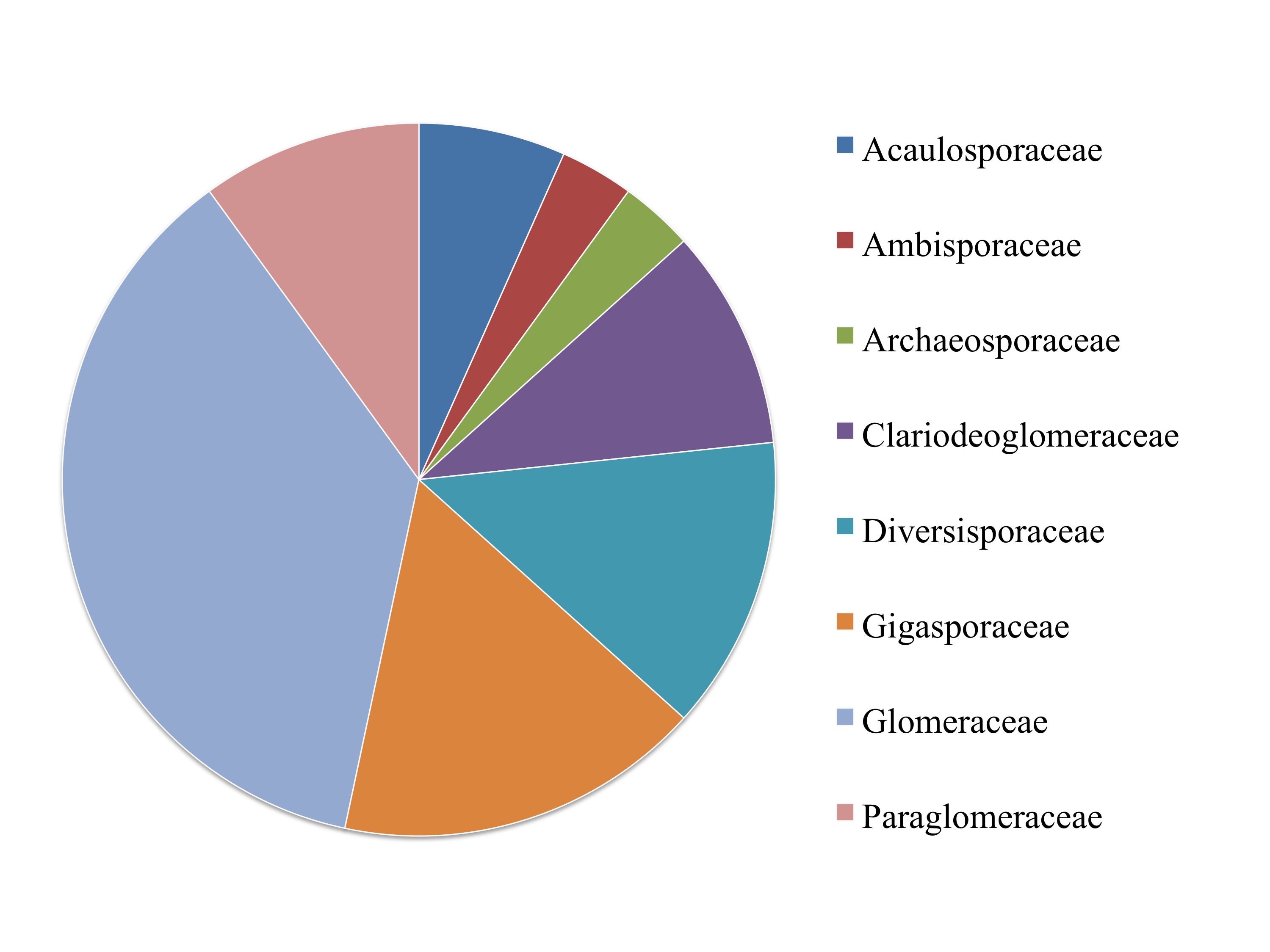


| Species Identified |
| --- |
| *Acaulospora lacunosa* |
| *Acaulospora sp.* |
| *Ambispora sp.* |
| *Archaeospora trappei* |
| *Claroideoglomus claroideum* |
| *Claroideoglomus lamellosum* |
| *Claroideoglomus luteum* |
| *Diversispora aurantia* |
| *Diversispora epigaea* |
| *Diversispora epigaea* |
| *Diversispora sp.* |
| *Gigaspora rosea* |
| *Scutellospora biornata* |
| *Scutellospora calospora* |
| *Scutellospora gregaria* |
| *Scutellospora sp.* |
| *Funneliformis caledonius* |
| *Funneliformis coronatus* |
| *Funneliformis fragilistratus* |
| *Funneliformis geosporus* |
| *Funneliformis verruculosus* |
| *Glomus albidum* |
| *Glomus indicum* |
| *Rhizophagus clarus* |
| *Rhizophagus fasciculatum* |
| *Rhizophagus intraradices* |
| *Rhizophagus manihotis* |
| *Paraglomus brasilianum* |
| *Paraglomus majewskii* |
| *Paraglomus occultum* |
| *Acaulospora lacunosa* |

**Table S2.** Three-way ANOVA table of the field experiment for AMF colonization.

| Source | Type III Sum of Squares | df | Mean Square | F | Sig. |
| --- | --- | --- | --- | --- | --- |
| Corrected Model | 7.6 | 23 | 0.3 | 5 | <0.001 |
| Site | 0.5 | 2 | 0.3 | 3.8 | 0.023 |
| Treatment | 1.2 | 1 | 1.2 | 17.8 | <0.001 |
| Species | 3.8 | 3 | 1.3 | 18.9 | <0.001 |
| Site * Treatment | 0.1 | 2 | 0.1 | 0.8 | 0.438 |
| Site * Species | 1.8 | 6 | 0.3 | 4.4 | <0.001 |
| Treatment * Species | 0.4 | 3 | 0.1 | 2 | 0.121 |
| Site*Treatment*Species | 0.4 | 6 | 0.1 | 1.1 | 0.392 |
| Error | 18.6 | 281 | 0.1 |  |  |
| Total | 26.2 | 304 |  |  |  |

**Table S3.** Three-way ANOVA table of the field experiment for plant biomass.

| Source | Type III Sum of Squares | df | Mean Square | F | Sig. |
| --- | --- | --- | --- | --- | --- |
| Corrected Model | 335.2 | 23 | 14.6 | 103.3 | <0.0001 |
| Site | 15.7 | 2 | 7.9 | 55.8 | <0.0001 |
| Treatment | 0.3 | 1 | 0.3 | 2.1 | 0.149 |
| Species | 193.5 | 3 | 64.5 | 457.1 | <0.0001 |
| Site * Treatment | 1.3 | 2 | 0.7 | 4.6 | 0.01 |
| Site * Species | 16.6 | 6 | 2.8 | 19.6 | <0.0001 |
| Treatment * Species | 1.5 | 3 | 0.5 | 3.5 | 0.016 |
| Site*Treatment*Species | 2.1 | 6 | 0.3 | 2.5 | 0.024 |
| Error | 40.9 | 290 | 0.1 |  |  |
| Total | 376.1 | 313 |  |  |  |

|  |  | **Soil Temp.** | **Water Avail.** | **Plant P** | **Plant N** | **Plant N:P** | **Soil P** | **Soil N** | **Soil N:P** | **Diversity** | **AM Col.** | **Biomass** |
| --- | --- | --- | --- | --- | --- | --- | --- | --- | --- | --- | --- | --- |
| **Site** |  | **(°C)** | **Matric ψ** | **wt%** | **wt%** | **wt%** | ***** | ***** | ***** | **(1/D)** | **%** | **(g)** |
| **Southern** |  |  |  |  |  |  |  |  |  |  |  |  |
| Control | **Mean**  (SD) | **14.6**  (1.4) | **-539.5**  (297.9) | **0.39**  (0.16) | **1.83**  (1.12) | **4.61**  (1.69) | **53.4**  (26) | **187.7**  (74.5) | **3.59**  (2.1) | **4.2**  (1.6) | **78.0**  (9.0) | **2.96**  (2.83) |
| Heated | **Mean**  (SD) | **16.7**  (2) | **-1247.4**  (426.2) | **0.24**  (0.09) | **1.77**  (0.51) | **7.49**  (1.62) | **32.9**  (5.3) | **233.4**  (55.5) | **6.93**  (1.21) | **2.22**  (0.76) | **59.0**  (10.0) | **3.87**  (0.75) |
| **Central** |  |  |  |  |  |  |  |  |  |  |  |  |
| Control | **Mean**  (SD) | **15.5**  (1.8) | **-170.9**  (72.1) | **0.17**  (0.05) | **1.95**  (1.38) | **10.33**  (1.79) | **7.5**  (2.8) | **7.2**  (7.6) | **0.69**  (2.35) | **6.85**  (1.76) | **68.0**  (7.0) | **2.35**  (3.45) |
| Heated | **Mean**  (SD) | **18.4**  (1.4) | **-272.8**  (232.4) | **0.19**  (0.06) | **2.12**  (1.9) | **9.31**  (2.18) | **14.6**  (5.7) | **9**  (5.8) | **0.55**  (2.97) | **6.43**  (1.09) | **57.0**  (9.0) | **3.47**  (5.35) |
| **Northern** |  |  |  |  |  |  |  |  |  |  |  |  |
| Control | **Mean**  (SD) | **17**  (1.6) | **-643.5**  (421.6) | **0.12**  (0.05) | **2.8**  (2.43) | **18.92**  (2.76) | **4.2**  (3.3) | **4.4**  (2.2) | **1.15**  (2.38) | **7.62**  (1.79) | **70.0**  (7.0) | **0.74**  (0.95) |
| Heated | **Mean**  (SD) | **20.2**  (1.7) | **-1193.2**  (529.6) | **0.13**  (0.04) | **3.13**  (2.89) | **18.27**  (2.29) | **2.4**  (0.7) | **3.8**  (1.6) | **1.55**  (1.67) | **7.69**  (0.61) | **63.0**  (7.0) | **1.31**  (1.11) |
| **Total** |  |  |  |  |  |  |  |  |  |  |  |  |
| Control | **Mean**  (SD) | **15.8**  (1.9) | **-442.6**  (366.4) | **0.21**  (0.14) | **2.22**  (1.82) | **10.28**  (2.54) | **18.8**  (25.3) | **55.4**  (90) | **1.3**  (2.88) | **6.4**  (2.21) | **0.72**  (0.08) | **1.95**  (2.78) |
| Heated | **Mean**  (SD) | **18.8**  (2.2) | **-861.6**  (617.2) | **0.17**  (0.08) | **2.47**  (2.27) | **11.75**  (2.31) | **13.3**  (12.2) | **53.2**  (95.7) | **1.44**  (3.28) | **6.09**  (2.23) | **0.6**  (0.08) | **2.64**  (4.24) |

**Table S4.** Means and standard deviations of the structural equation model variables used to test the three *a priori* models.

***** units of soil N and soil P: μg/10cm^2^/April-July.

**Table S5.** Pearson’s correlations of the structural equation model variables. Boldface represents P < 0.01, italicized represents P < 0.05.

| **Correlations** | **AM Col %** | **Biomass** | **Soil N** | **Soil P** | **Soil N:P** | **Plant N:P** | **Plant N** | **Plant P** | **Soil Temp** | **Water Avail.** |
| --- | --- | --- | --- | --- | --- | --- | --- | --- | --- | --- |
| **AM Col.** | 1.00 |  |  |  |  |  |  |  |  |  |
| **Biomass** | 0.05 | 1.00 |  |  |  |  |  |  |  |  |
| **Soil N** | 0.09 | **0.25** | 1.00 |  |  |  |  |  |  |  |
| **Soil P** | 0.04 | **0.28** | **0.77** | 1.00 |  |  |  |  |  |  |
| **Soil N:P** | 0.10 | 0.10 | **0.74** | *0.13* | 1.00 |  |  |  |  |  |
| **Plant N:P** | -0.03 | **-0.55** | **-0.42** | **-0.50** | *-0.13* | 1.00 |  |  |  |  |
| **Plant N** | 0.03 | -**0.57** | *-0.13* | *-0.14* | -0.04 | **0.83** | 1.00 |  |  |  |
| **Plant P** | 0.09 | **0.27** | **0.61** | **0.68** | **0.23** | **-0.66** | **-0.17** | 1.00 |  |  |
| **Soil Temp** | **-0.23** | **0.39** | **-0.38** | **-0.47** | -0.10 | -0.02 | **-0.27** | **-0.30** | 1.00 |  |
| **Water Avail.** | 0.07 | **-0.41** | **-0.16** | *-0.12* | **-0.37** | **0.19** | **0.32** | -0.01 | **-0.54** | 1.00 |
| **Diversity** | -0.02 | **-0.17** | **-0.80** | **-0.50** | **-0.64** | **-0.34** | *0.13* | **-0.42** | **0.26** | **0.32** |

**Table S6.** Standardized direct, indirect, and total effects of the southern site only structural equation model.

| Effect of Variable 1 | on | Variable 2 | Direct Effect | Indirect Effect | Total Effect |
| --- | --- | --- | --- | --- | --- |
| Soil Temperature | 🡪 | Soil Water Availability | -0.77 | N/A | -0.77 |
| Soil Temperature | 🡪 | Soil N:P | 0.62 | -0.20 | 0.42 |
| Soil Temperature | 🡪 | Plant Diversity | -0.11 | -0.28 | -0.39 |
| Soil Temperature | 🡪 | AMF colonization | -0.41 | 0.04 | -0.37 |
| Soil Temperature | 🡪 | Plant N:P | N/A | 0.25 | 0.25 |
| Soil Temperature | 🡪 | Plant Biomass | 0.50 | -0.11 | 0.39 |
| Soil Water Availability | 🡪 | Soil N:P | 0.26 | N/A | 0.26 |
| Soil Water Availability | 🡪 | Plant Diversity | -0.11 | -0.23 | -0.34 |
| Soil Water Availability | 🡪 | AMF colonization | 0.05 | 0.02 | 0.07 |
| Soil Water Availability | 🡪 | Plant N:P | -0.13 | 0.14 | 0.01 |
| Soil Water Availability | 🡪 | Plant Biomass | -0.24 | -0.07 | -0.31 |
| Soil N:P | 🡪 | Plant Diversity | -0.88 | N/A | -0.88 |
| Soil N:P | 🡪 | AMF colonization | 0.38 | -0.20 | 0.18 |
| Soil N:P | 🡪 | Plant N:P | -0.20 | 0.49 | 0.29 |
| Soil N:P | 🡪 | Plant Biomass | -0.17 | -0.04 | -0.21 |
| Plant Diversity | 🡪 | AMF colonization | 0.23 | N/A | 0.23 |
| Plant Diversity | 🡪 | Plant N:P | -0.56 | -0.01 | -0.57 |
| Plant Diversity | 🡪 | Plant Biomass | 0.17 | 0.06 | 0.23 |
| AMF colonization | 🡪 | Plant N:P | -0.03 | N/A | -0.03 |
| AMF colonization | 🡪 | Plant Biomass | 0.48 | 0.00 | 0.48 |
| Plant N:P | 🡪 | Plant Biomass | 0.10 | N/A | 0.10 |

**Supporting Information Tables S7-S12:** Soil and plant nutrient results.

*Soil Nutrient Data*

Soil P availability, N availability and their ratio differed among sites, with the southern site having nearly 10-fold higher nutrient availability (Figure S1). The effect of the heating treatment depended on site (Tables S7-S9). Heating decreased P availability in the southern and northern sites but increased it in the central site (*P* < 0.0001). Heating increased N availability in the southern and central sites (*P* < 0.004) with no effect in the northern site. Heating also increased the N:P ratio in the southern and northern sites (*P* < 0.023), suggesting a shift toward P limitation in response to warming.

**Table S7.** Two-way ANOVA table of soil P availability.

| Source | Type III Sum of Squares | df | Mean Square | F | Sig. |
| --- | --- | --- | --- | --- | --- |
| Corrected Model | 68.6 | 5 | 13.7 | 374.0 | <0.0001 |
| Site | 61.6 | 2 | 30.8 | 839.6 | <0.0001 |
| Treatment | 0.0 | 1 | 0.0 | 0.8 | 0.4 |
| Site * Treatment | 3.7 | 2 | 1.8 | 50.0 | <0.0001 |
| Error | 11.3 | 308 | 0.0 |  |  |
| Total | 79.9 | 313 |  |  |  |

**Table S8.** Two-way ANOVA table of soil N availability

| Source | Type III Sum of Squares | df | Mean Square | F | Sig. |
| --- | --- | --- | --- | --- | --- |
| Corrected Model | 154.7 | 5 | 30.9 | 478.1 | <0.0001 |
| Site | 150.0 | 2 | 75.0 | 1159.3 | <0.0001 |
| Treatment | 0.5 | 1 | 0.5 | 8.1 | 0.005 |
| Site * Treatment | 0.8 | 2 | 0.4 | 6.1 | 0.002 |
| Error | 19.9 | 308 | 0.1 |  |  |
| Total | 174.6 | 313 |  |  |  |

**Table S9.** Two-way ANOVA table of soil N:P ratio.

| Source | Type III Sum of Squares | df | Mean Square | F | Sig. |
| --- | --- | --- | --- | --- | --- |
| Corrected Model | 37.3 | 5 | 7.5 | 62.9 | <0.0001 |
| Site | 36.7 | 2 | 18.4 | 154.6 | <0.0001 |
| Treatment | 0.8 | 1 | 0.8 | 6.8 | 0.009 |
| Site * Treatment | 1.8 | 2 | 0.9 | 7.5 | 0.001 |
| Error | 36.6 | 308 | 0.1 |  |  |
| Total | 73.9 | 313 |  |  |  |

*Plant Nutrient Data*

Plant P content differed among sites and species, and these were interdependent (*P* < 0.0001, Table S10). Plant P content was highest in the southern site for all three perennial species, with a similar trend in the annual species, *Plectritis*. The effect of the heating treatment depended on site (*P* < 0.0001), and there was a marginal three-way interaction among treatment, site, and species (*P* = 0.09). The heating treatment decreased P content only in the southern site for *Eriophyllum* and *Prunella* (*P* < 0.013, Appendix A: Figure S2.A).

Plant N content differed by site and species, and they were interdependent (*P* < 0.0001, Table S11). There were no direct or interactive effects of heating (Appendix A: Figure S2.B). All species had relatively constant levels across sites and species, with the exception of *Plectritis*, which showed a dramatic increase in N from south to north.

The plant N:P ratio differed by site and species, and they were interdependent (*P* < 0.0001, Table S12). The plant N:P ratio differed among species but generally plants tended to have lower N:P ratios in the southern site (*P* < 0.0001, Appendix A: Figure S2.C). Plants with a ratio < 10 and > 20 are considered to be N limited and P limited, respectively (Güsewell, 2004). By these criteria, plants appear to be generally N limited or co-limited by the two nutrients. The effect of the heating treatment depended on site (*P* = 0.01), where heating increased the N:P ratio in the southern site (P < 0.1).

**Table S10.** Three-way ANOVA table of plant P content.

| Source | Type III Sum of Squares | df | Mean Square | F | Sig. |
| --- | --- | --- | --- | --- | --- |
| Corrected Model | 0.34 | 23 | 0.015 | 29.0 | <0.0001 |
| Site | 0.177 | 2 | 0.088 | 172.8 | <0.0001 |
| Treatment | 0.008 | 1 | 0.008 | 16.2 | <0.0001 |
| Species | 0.025 | 3 | 0.008 | 16.5 | <0.0001 |
| Site * Treatment | 0.023 | 2 | 0.012 | 22.6 | <0.0001 |
| Site * Species | 0.018 | 6 | 0.003 | 5.9 | <0.0001 |
| Treatment * Species | 0.002 | 3 | 0.001 | 1.0 | 0.37 |
| Site*Treatment*Species | 0.006 | 6 | 0.001 | 1.9 | 0.09 |
| Error | 0.148 | 290 | 0.001 |  |  |
| Total | 0.488 | 313 |  |  |  |

**Table S11.** Three-way ANOVA table of plant N content.

| Source | Type III Sum of Squares | df | Mean Square | F | Sig. |
| --- | --- | --- | --- | --- | --- |
| Corrected Model | 7.985 | 23 | 0.347 | 27.8 | <0.0001 |
| Site | 0.398 | 2 | 0.199 | 15.9 | <0.0001 |
| Treatment | 0.002 | 1 | 0.002 | 0.1 | 0.72 |
| Species | 2.183 | 3 | 0.728 | 58.2 | <0.0001 |
| Site * Treatment | 0.01 | 2 | 0.005 | 0.4 | 0.66 |
| Site * Species | 2.745 | 6 | 0.457 | 36.6 | <0.0001 |
| Treatment * Species | 0.011 | 3 | 0.004 | 0.3 | 0.83 |
| Site*Treatment*Species | 0.072 | 6 | 0.012 | 1.0 | 0.46 |
| Error | 3.589 | 287 | 0.013 |  |  |
| Total | 11.573 | 310 |  |  |  |

**Table S12.** Three-way ANOVA table of plant N:P ratio.

| Source | Type III Sum of Squares | df | Mean Square | F | Sig. |
| --- | --- | --- | --- | --- | --- |
| Corrected Model | 33.303 | 23 | 1.448 | 31.5 | <0.0001 |
| Site | 10.539 | 2 | 5.269 | 114.7 | <0.0001 |
| Treatment | 0.079 | 1 | 0.079 | 1.7 | 0.19 |
| Species | 5.39 | 3 | 1.797 | 39.1 | <0.0001 |
| Site * Treatment | 0.401 | 2 | 0.201 | 4.4 | 0.01 |
| Site * Species | 7.098 | 6 | 1.183 | 25.8 | <0.0001 |
| Treatment * Species | 0.177 | 3 | 0.059 | 1.3 | 0.28 |
| Site*Treatment*Species | 0.306 | 6 | 0.051 | 1.1 | 0.36 |
| Error | 13.181 | 287 | 0.046 |  |  |
| Corrected Total | 46.484 | 310 |  |  |  |
